# Evaluation of in vivo anti-malarial potential of *omidun* obtained from fermented maize in Ibadan, Nigeria

**DOI:** 10.1186/s12936-020-03486-0

**Published:** 2020-11-19

**Authors:** Favour O. Omeiza, George O. Ademowo, Funmilola A. Ayeni

**Affiliations:** 1 Department of Pharmaceutical Microbiology, Faculty of Pharmacy, University of Ibadan, Ibadan, Nigeria; 2 Institute for Advanced Medical Research and Training, College of Medicine, University of Ibadan, Ibadan, Nigeria

**Keywords:** *Omidun*, *Plasmodium berghei*, Curative, Suppressive, Prophylactic

## Abstract

**Background:**

The menace of resistance to anti-malarial drugs is a great challenge to malaria control, necessitating the search for new anti-malarial agents. This search has led to the exploration of natural products for efficacy in malaria therapy. *Omidun* is the supernatant of fermenting maize (*ogi*) slurry that has been widely investigated and reported to possess several health benefits and it is used traditionally as solvent for preparing anti-malarial herbs. However, there is no information on the anti-malarial activity of *omidun* itself. This study was conducted to investigate the prophylactic, curative and suppressive anti-malarial potential of *omidun.*

**Methods:**

Experimental mice in the curative group were infected with 1 × 10^6^ cells of *Plasmodium berghei* strain ANKA and treated with either 0.2 ml of *omidun* containing 3 × 10^9^ cfu/ml of viable lactic acid bacteria or 0.2 ml of 5 mg/kg of chloroquine (positive control) or 0.2 ml of saline (negative control) for 4 days from day 3 post infection. The prophylactic group of mice were pre-treated with either *omidun*, chloroquine or saline for 4 days before infection with *P. berghei*, while the suppressive group was treated with *omidun* or chloroquine or saline and infected with *P. berghei* simultaneously. A group of mice were uninfected but treated (with *omidun* and control samples), while a final group was uninfected and untreated (controls). Parasitaemia and histopathology analysis were done in all groups.

**Results:**

The curative and suppressive groups showed a significant difference between the *omidun*-treated mice (100% parasitaemia reduction) and the untreated mice (54.5% parasitaemia increase). There was no significance difference between the *omidun* treatment and chloroquine (positive control) treatment in suppressive group as both treatment had 100% parasitaemia reduction. The *omidun* prophylactic treatment however did not show any parasitaemia suppression, but a significant difference was observed between the *omidun* treatment (85% increase) and the chloroquine (positive control) treatment (100% reduction) in the group. *Omidun* treatment is non-toxic to the kidney.

**Conclusion:**

This study provides scientific evidence supporting *omidun* usage in the treatment of malaria. Consequently, further work may yield the specific component of *omidun* responsible for the anti-malarial activity.

## Background

One of the most important infectious diseases globally is malaria, which is a tropical disease caused by protozoan parasites that belongs to the genus *Plasmodium* and are transmitted by mosquitoes [1]. It poses a major threat to world public health with about 219 million people being infected in the world and about 435,000 deaths occurring annually [2]. In Africa and even globally, about 10 and 3% of disability-adjusted life years results from malaria mortality, respectively [3].

The alarming increase in anti-malarial drug resistance calls for an intense search for alternative anti-malarial agents [4] that are safe, inexpensive and readily available to people, particularly those in the developing countries. The communities in endemic areas continually search for malaria remedies in natural products [5]. Herbal formulations have been in use for thousands of years and have proved effective to a very large extent over the years. In addition to being effective, herbal medicines have the advantage of being readily available and relatively cheap compared to western medicine. In Nigeria, the traditional medical source may be herbs, leaves and tree bark of certain plants, soaked in solvents such as water, alcohol, palm wine, and supernatant (*omidun*) of fermenting maize slurry (*ogi*). These solvents, of which the most preferred have been found to be *omidun* and palm wine, are believed to extract the active ingredients in the plant parts [6].

*Omidun* is reported to contain a large number of lactic acid bacteria (LAB) accounting for its efficiency in the treatment of diarrhoea [7]. Traditionally, in Nigeria and predominantly in the southwestern region, *omidun* has been reported to be of great medicinal relevance [8]. The root bark of certain plants has been soaked in *omidun* and used to treat malaria and fever; it is also commonly used as solvent for extracting herbs, removing stains on dishes and killing insects [8]. It has been administered in the southwestern part of Nigeria to people suffering from gastrointestinal disorder to minimize discomfort [9]. There are reports that *omidun* possesses LAB that have inhibitory activities against pathogenic *Escherichia coli* [7, 8, 10, 11] and also antibacterial [12], antiviral [13] and anticolitic [14] properties. However, there is no information on anti-malarial properties of *omidun* when solely used. This study investigates anti-malarial properties of *omidun* in a mice model.

## Methods

### Preparation of *Omidun*

*Omidun* was prepared according to standard traditional method of preparation as previously reported [8]. The yellow variety of maize cereal grain was obtained from Bodija market, Ibadan, Nigeria, washed properly and 400 g was soaked in 600 ml of distilled water for 72 h at room temperature. The water was decanted and the grain transferred to a clean grinding machine for wet milling. The ground cereal was sieved with sterile muslin cloths, the filtrate was collected into a sterile container while the pomace was discarded. The filtrate was allowed to sediment for 3 days during which fermentation occurred. The supernatant (top water) *omidun* was collected while slightly scraping the surface of sediment (*ogi*) to fully obtain LAB that might have settled on its surface [12]. The collected supernatant and the slightly scraped sediments were pooled together to obtain a uniform mixture. The *omidun* was used within 6 days of milling (day 3 to day 6) after which a fresh batch was prepared. The LAB in *omidun* with lightly scrapped *ogi* surface was quantified by viable count technique [12].

### In vivo pharmacological studies

Experimental animals and parasites Eight to 12 weeks old Swiss albino mice, weighing an average of 20 g (weight ≥ 18– ≤ 24) were acquired and acclimatized for 2 weeks. They were maintained at a room temperature of about 25 °C and 12:12 light/dark cycle, with food and water given ad libitum. All experiments were conducted in accordance with internationally accepted laboratory animal use, care and guidelines. Ethical approval for the study was obtained from Animal Care and Use Research Ethics Committee, (Reference number: UI-ACUREC/18/0142) University of Ibadan, Ibadan on 3 June, 2019.

*Plasmodium berghei* strain ANKA was the experimental parasite used and it was obtained from the Institute for Medical Research and Training (IMRAT), University College Hospital, University of Ibadan. This parasite was established and maintained by regular passage for the purpose of this experiment.

### Grouping and dosing of animals

For each model (curative, suppressive, prophylactic) group, 15 mice were grouped into 3 groups of 5 mice each. Group I mice were treated with *omidun* (0.2 ml of 3 × 10^9^ cfu/ml of viable LAB cells), group II mice were treated with the standard drug (chloroquine, 0.2 ml of 5 mg/kg, positive control), while group III mice were treated with normal saline (negative control) (Table 1). Infections were done by intraperitoneal injection of 1 × 10^6^ cells of *P. berghei*. Each treatment was administered through oral route using oral gavage to ensure safe ingestion of the preparations.

**Table 1:** Experimental groups

Experimental groups
Curative groups
Infected, *omidun* treatment
Infected, chloroquine treatment–positive control
Infected, no treatment–negative control
**Prophylactic groups**
4 days prophylactic *omidun* treatment before infection
4 days prophylactic chloroquine treatment before infection–positive control
4 days prophylactic normal saline treatment before infection- negative control
**Suppressive groups**
Infection and *omidun* treatment introduced simultaneously
Infection and chloroquine introduced simultaneously–positive control
Infection and normal saline introduced simultaneously- negative control
**No Infection, treatment groups**
No Infection, *omidun* Treatment
No infection, chloroquine treatment
**No infection, no treatment group**
Uninfected and untreated mice

### Curative test

The mice in this group were infected with *P. berghei*. Blood smear microscopy was carried out for each animal to monitor parasitaemia from after 72 h of infection. From post-infection day 3, they were treated for 4 days by oral administration of *omidun* or chloroquine. The negative group was infected but no treatment was administered even after post-infection day 3.

### Prophylactic test

The mice in this group were treated with 4 days oral administration of *omidun* or 4 days oral administration of chloroquine. Then they were infected with *P. berghei*. Blood smear microscopy was carried out for each animal to monitor parasitaemia from 72 h post infection till post infection day 4.

### Suppressive test

The mice in this group were infected by intra-peritoneal injection of *P. berghei* and oral administration of either *omidun* or chloroquine or saline samples simultaneously. From post-infection day 3, there was continuous administration of *omidun* or chloroquine or saline for 4 days. Blood smear microscopy was carried out for each animal to check for suppressive potentials of *omidun* from 72 h post-infection until post-infection day 4.

### Blood smear preparation and parasitaemia determination

Blood smear microscopy was carried out by microscopic examination of Giemsa stained smears of thin films of blood from animals. Smears were made from the tails of each mouse and prepared into microscopic slides. These stained smears were then microscopically examined and parasitaemia was scored as follows:

$$Parasitaemia\;\left( {\% } \right)\;\text{ = }\;\frac{{Number\;of\;parasitized\;red\;blood\;cells\;\left( {pRBCs} \right)}}{{Total\;number\;of\;\;red\;blood\;cells\;\left( {totalRBCs} \right)}}\; \times 100$$

### Animal euthanasia

The animals were observed for a week after which they were euthanized. The animals to be euthanized were first anaesthetized with ketamine given subcutaneously. The animals were euthanized by intra-cardiac injection of sodium pentobarbital (100 mg/kg) using 25G needle. All procedures were carried out to avoid causing unnecessary pain to animals, in accordance to the rules of animal care and use research.

### Histopathological examination

Animals whose organs needed to be examined for extent of damage to organs by the induced malaria infection were painlessly euthanized as highlighted above and the required organs, i.e., liver and kidney were harvested. The harvested organs were fixed in 10% (vol/vol) neutral buffered formalin, and further embedded in paraffin, then sectioned with a microtome into 5 to 7-μm thick paraffin slices. These slices were dewaxed and stained with haematoxylin and eosin stains. The stained tissues were dehydrated with 70% ethanol, followed by 90% ethanol and two changes of 100% ethanol for 3 min each. Then they were cleaned with two changes of xylene for 3 min after which they were cover-slipped. The microscopy was carried out using the × 40 objective lens and pathological observations were recorded.

### Statistical analysis

Statistical analysis was done using the analysis of variance between groups (ANOVA) to compare difference in percentage inhibition of parasite growth at p < 0.05.

## Results

### Efficacy analysis of *Omidun*

**Fig. 1:**
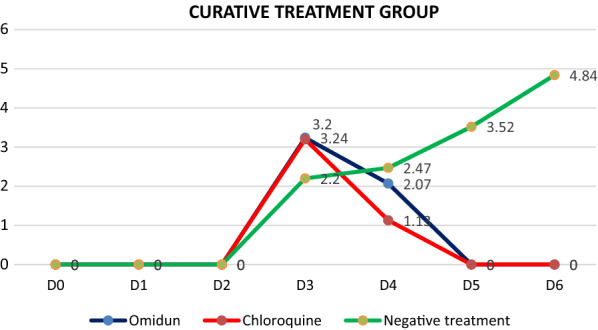
Parasitaemia values of the curative group

The mean parasitaemia values of the *omidun* curative study was found to be 3.24% on day 3 post infection; this value dropped to 2.07% (36.1% decrease) after 24 h (day 4), 0.18% (94.4% decrease) after 48 h (day 5) and a final value of 0% (100% decrease) after 72 h (day 6). The chloroquine treatment group (positive control) showed a mean parasitaemia value of 3.20% on day 3 post infection; this value dropped to 1.13% (64.7% decrease) after 24 h and further decreased to 0% (100% decrease) after 48 h. The mean parasitaemia values in the negative control (infected but not treated) was found to be 2.20% on day 3 post infection, this value increased to 2.47% (10.9% increase) after 24 h, 3.52% (37.5% increase) after 48 h, and a further increase to 4.84% (54.5%) after 72 h. The curative study revealed that *omidun* exerted 100% parasitaemia clearance after 72 h while chloroquine treatment exerted 100% parasitaemia clearance after 48 h. The parasitaemia level for the untreated group increased by 54.5% after 72 h post-infection day (Fig. 1).

**Fig. 2:**
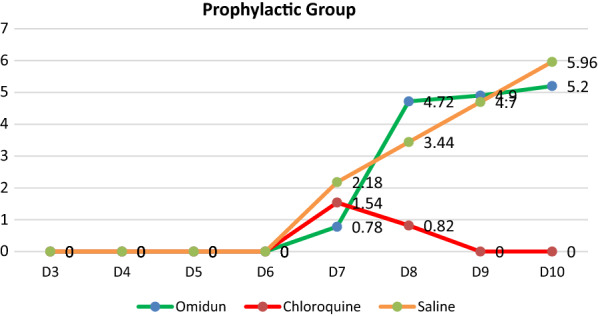
Parasitaemia values of the prophylactic group

The *omidun* 4-days prophylactic treatment group had a very low mean parasitaemia value of 0.78% on day 3 post infection, this value drastically increased to 4.72% (83.5% increase) after 24 h, 4.9% (84.1% increase) after 48 h and further increased to 5.2% (85% increase after 72 h. The chloroquine 4-days prophylactic treatment group showed a low mean parasitaemia value of 1.54% on day 3 post infection which decreased to 0.82% (46.8% decrease) after 24 h and finally dropped to 0% (100% decrease) after 48 h. The negative control group (prophylactic) showed a mean parasitaemia value of 2.18% on day 3 post infection and consistently increased to 5.96% (63.4% increase) after 72 h. The prophylactic study revealed that *omidun* helped in giving a very low parasitaemia value on day 3 post infection but was unable to prevent the rise in the parasitaemia level for subsequent days; the chloroquine treatment was however able to exert a 100% clearance of the parasite after 48 h. The negative control prophylactic treatment had a consistent rise up to 63.4% increase after 72 h (Fig. 2).

**Fig. 3:**
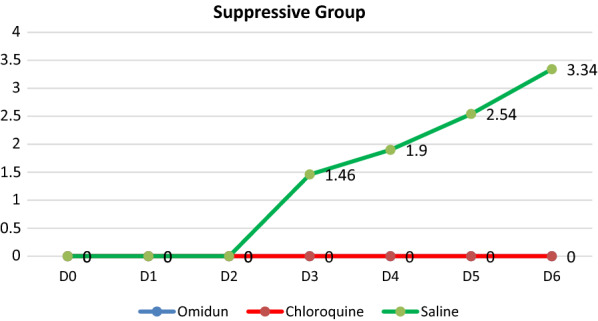
Parasitaemia value for suppressive group

The antiplasmodial study for the suppressive group showed that there was no visible parasite in the blood stream of the animals in the *omidun* treatment group and also in the chloroquine treatment group. Parasites were however seen in the group treated with normal saline and the parasitaemia level increased from 1.46 to 3.34% (56.3% increase) between days 3 and 6. The total absence of parasites in the suppressive group for the *omidun* treatment revealed that the simultaneous administration of the *omidun* and the parasite and the continuous treatment with *omidun* hindered the appearance of the parasite in the blood stream, consistent with the positive control group and in contrast to the negative control group (Fig. 3).

There was significant difference between the *omidun*-treated mice and the untreated mice (negative control) and there was no significance difference between the *omidun* treatment and the chloroquine (positive control treatment for the curative and suppressive groups with p value less than 0.05 (p < 0.05) (Tables 2, 3, and 4).

**Table 2:** ANOVA analysis of the curative group

Source	Sum of squares	Df	Mean of squares	F	Prob > F
Model	6.3755	2	3.1878	1.37	0.2791
Treatment	6.3755	2	3.1878	1.37	0.2791
Residual	41.8501	18	2.3250		
Total	48.2257	20	2.4112

**Table 3:** ANOVA analysis of the prophylactic group

Source	Sum of squares	DF	Mean of squares	F	Prob > F
Model	14.6081	2	7.3040	3.18	0.0621
Treatment	14.6081	2	7.3040	3.18	0.0621
Residual	48.2124	21	2.2958		
Total	62.8205	23	2.7313

**Table 4:** ANOVA analysis of the suppressive group

Source	Sum of squares	Df	Mean of squares	F	Prob > F
Model	8.0609	2	4.0305	6.60	0.0071
Treatment	8.0609	2	4.0305	6.60	0.0071
Residual	10.9918	18	0.6106		
Total	19.0527	20	0.9526		

### Safety analysis of *Omidun*

The liver pathology of *omidun* curative treatment group had severe diffuse cord atrophy while that of the chloroquine curative treatment group showed moderate centrilobular hepatocellular vacuolar degeneration. The liver pathology of the infected but not treated group shows severe diffuse cord atrophy and hepatocellular necrosis. The kidney pathology of the curative treatment group reveals that there was no observable lesion in the *omidun* treatment while that of the chloroquine curative treatment had tubular epithelial degeneration and necrosis. The kidney pathology of the infected but not treated group showed tubular epithelial necrosis and inflammation (Table 5).

**Table 5:**
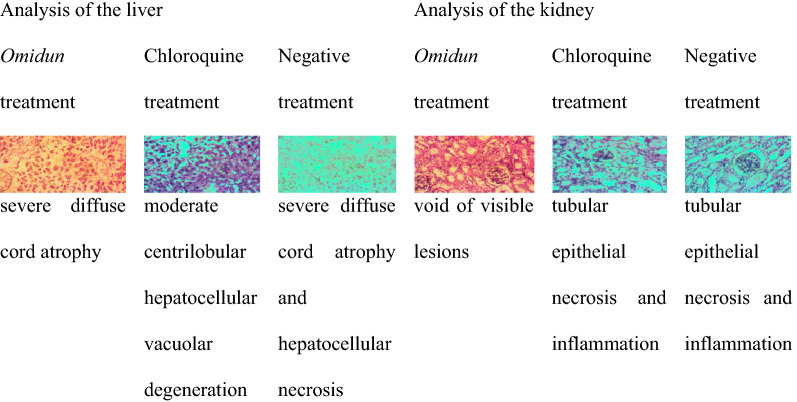
Histopathological observation of the liver and kidney for the curative group

In the prophylactic group, the *omidun* treatment revealed moderate diffuse cord atrophy and centrilobular coagulation necrosis in the liver while the kidney had necrosis of tubular epithelial cells and interstitial inflammation. The chloroquine treatment revealed moderate centrilobular hepatocellular atrophy and accentuation of sinusoids in the liver while the kidney had patchy tubular epithelial coagulation necrosis (Table 6).

**Table 6:**
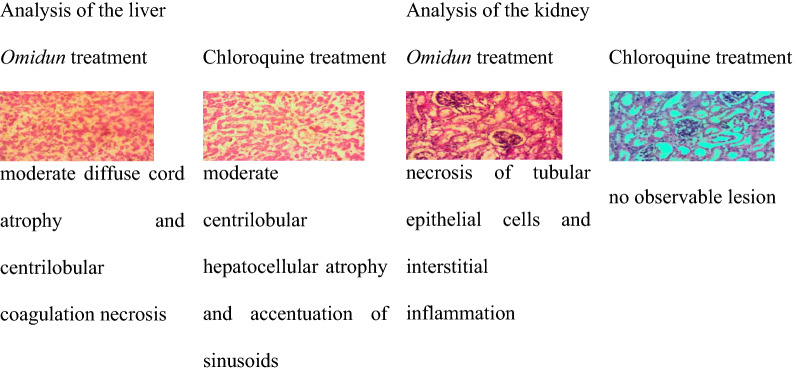
Histopathological observation of the liver and kidney for the prophylactic group

In the suppressive group, *omidun* treatment revealed diffuse atrophy of hepatocytes and cord in the liver while the kidney of the animal had no observable lesion. The chloroquine treatment revealed moderate diffuse hepatocellular atrophy and accentuation of sinusoids in the liver of the animal while the kidney had necrosis of tubular epithelial cells and interstitial inflammation (Table 7).

**Table 7:**
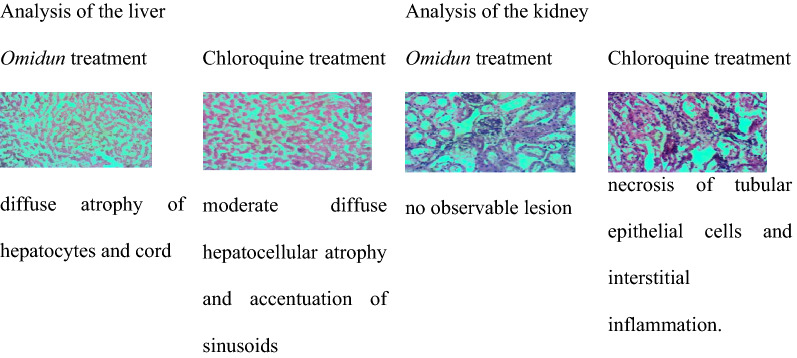
Histopathological observation of the liver and kidney for the suppressive group

The uninfected group but administered with chloroquine had severe diffuse cord atrophy and hepatocellular necrosis in the liver with tubular epithelial necrosis and inflammation in the kidney. The uninfected group but administered with *omidun* showed that the liver had random hepatocellular vacuolar degeneration while the kidney had no visible lesion. The uninfected and untreated group showed that the liver had moderate diffuse vacuolar degeneration of the hepatocytes while the kidney had no visible lesion (Table 8).

**Table 8:**
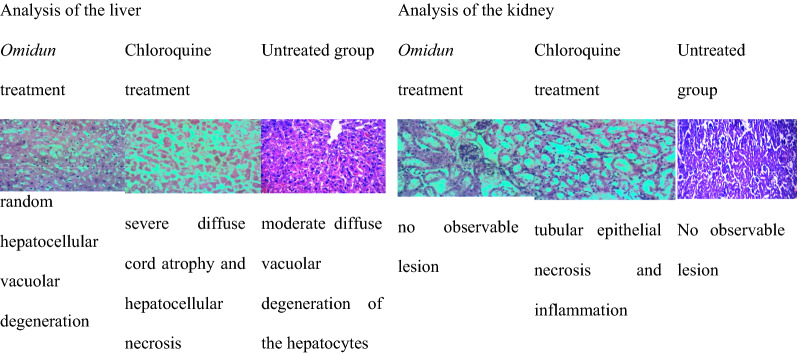
Histopathological observation of the liver and kidney for the uninfected group

## Discussion

### Efficacy

The anti-malarial activity of *omidun*, used in traditional medicine in Nigeria and elsewhere in suppressive, curative and prophylactic mice test models are reported. The study revealed a good parasitaemia reduction in the curative and suppressive group while the prophylactic group had no reduction in parasitaemia values. This is indicative of the high anti-malarial potentials of *omidun*, which is also greatly dependent on its usage.

The pattern of reduction in the parasitaemia levels of the *omidun* curative group is consistent with the positive control group where chloroquine was administered and in contrast to the negative control where no treatment was administered, causing a consistent increase in the parasitaemia levels of the animals. The curative *omidun* treatment shows a significant difference statistically (p < 0.05) with that of the negative control groups while showing no statistical significant difference with the positive control group, chloroquine, i.e., the effect of *omidun* is highly similar to that of chloroquine but differs greatly from the untreated group. This is similar to the study carried out by Berthi et al. [15] on the anti-plasmodial effect of plant extracts from *Picrolemma huberi* and *Picramnia latifolia* where *Picrolemma huberi* had a good reduction in parasitaemia levels consistent with the chloroquine treatment, which was also used as the positive control.

In the prophylactic study, it was observed that the parasitaemia level of the *omidun* group on the 5th day (day 4) immediately after the prophylaxis administration was very low (0.78%) compared to that of the positive (1.54%) and negative control (2.18%); this suggests that the *omidun* was able to prevent the appearance of the parasite in the blood. However, the parasitaemia level of the test group increased greatly on the 8th day (day 7) due to stoppage in administration of the *omidun,* suggesting that the LAB and its metabolites present in the *omidun* might have been cleared from the animal, thus preventing the anti-malarial effect to remain and accounting for the rise in the parasitaemia level. This is similar to the study by Misganaw et al. [16] on the evaluation of the anti-malarial activity of crude extract and solvent fractions of the leaves of *Olea europaea (Oleaceae)* in mice where it was observed that in the prophylactic test, the extract produced the lowest percentage suppression of parasitaemia compared to its effect in the curative and suppressive group, which however demonstrated significant suppressive effect on the level of parasitaemia compared to the negative control group. This finding is in agreement with the studies carried out by Unekwuojo et al. [17] on the suppressive, curative and prophylactic potentials of *Morinda lucida* (Benth) against erythrocytic stage of mice infected with chloroquine-sensitive *P. berghei* where the prophylactic treatment had the lowest parasitaemia reduction. It can thus be said that the effectiveness of the *omidun* lies in its continuous use until the parasite is completely eradicated.

The suppressive treatment of *omidun* revealed that there was total suppression of parasites in the blood stream of the animals just as was also observed in the chloroquine treatment group. Parasites were however seen in the group treated with normal saline (negative control) where the parasitaemia level increased from 1.46 to 3.34%. This suppressive *omidun* treatment shows a statistical significant difference with that of the negative control groups while showing no statistical significance with the positive control group. This means that the effect of *omidun* is highly similar to that of chloroquine but differs greatly from the untreated group. This is similar to the result obtained by Godwin et al. [18] in the anti-malarial study of *Verbena hastate.* The total absence of parasite in the suppressive group for the *omidun* treatment revealed that the simultaneous administration of the *omidun* and the parasite and the continuous treatment with *omidun* hindered the appearance of the parasite in the blood stream consistent with the positive control and in contrast to the negative control. A similar result was reported by Jackie et al. [19] where *Croton macrostachyus* stem bark ethyl extract had 100% chemoprotective activity against malaria. This implies that *Omidun* is efficacious in suppressing the appearance and survival of malarial parasite in the blood stream when there is a regular and continuous usage.

The *omidun* curative and suppressive treatment was found to exert 100% parasite clearance from the blood stream of animals, it can therefore be said that *omidun* has very good anti-malarial activity. This anti-malarial activity was classified according to Rasoanaivo et al. [20] classification which states that extracts with very good or good activity should have 90–100% parasitaemia inhibition, extracts with good to moderate activity should have 50–90% inhibition, extract with moderate to weak activity should have 10–50% inhibition and extracts that are inactive have 0% inhibition.

This observed anti-malarial activity of *omidun* in the curative and suppressive group is consistent with the traditional use of this liquid as a solvent for herbal medications against malaria and indicative of its potential as a chemotherapeutic anti-malarial agent even when used alone without herbal compounds. The anti-malarial activity of *omidun* may be attributed to the high level of LAB present in it with their various metabolites [7], which have been shown to have rich nutritional benefits, health values, antimicrobial activity, and even antiplasmodial potentials [21].

LAB synthesizes antimicrobial peptides (AMPs), generally known as bacteriocins, to enable successful existence and enhance protection against pathogens [22, 23]. AMPs are showing potential as powerful weapons against bacteria, fungi and parasites considering their inhibitory spectrum. AMPs LR14 is a multi-peptide bacteriocin that has been purified from *Lactobaccilus plantarum* and has been investigated for activity against human pathogen *Plasmodium falciparum* [22].

Krugliak et al. [24] reported the anti-malarial effects of C18 fatty acids on *P. falciparum, Plasmodium yoelii nigeriensis* and on *Plasmodium vinckei petteri* in vivo, stating that the C18 acids displayed a considerable and rapid inhibition of these parasites. Soh et al. [25] showed that ellagic acid had high activity in vitro against all *P. falciparum* strains regardless of their level of chloroquine and mefloquine resistance. The authors further reported that this acid was active in vivo against *P. vinckei petteri* in suppressive, curative and prophylactic murine tests without any toxicity. Moneriz et al. [26] reported the parasitostatic effect of maslinic acid.

It is important to note that the many properties of *omidun* could also be linked to the important contribution of LAB present in *omidun* with their metabolites. LAB possess the ability to boost the immune system and decrease the danger of diseases [27, 28] and to assist quicker recovery from sickness [29]. In addition, fermented foods such as *omidun* contain vitamin C, iron or zinc, which have been shown to help strengthen the immune system [30]. LAB also produce organic acids in the course of growing which are secreted into the surroundings and they possess antimicrobial properties [31].

### Safety

The liver is highly essential in the assessment of internal damages due to malaria infections. This is because the liver plays a vital role in the survival of *Plasmodium* cells [32]. It is highly important that a good anti-malarial agent possesses the ability to completely clear parasites from the liver and aid the immune system in conducting necessary internal repairs in a short time [32]. The *omidun* treatment group was found to be void of visible lesions in the kidney for the curative group but with severe diffuse cord atrophy in the liver. This shows that *omidun* treatment was completely unable to ameliorate damage done in the liver by the parasite, but it had little ameliorating effect as there was no necrosis recorded, in contrast to the untreated group that recorded severe diffuse cord atrophy and hepatocellular necrosis. However, no visible damage was observed in the kidney of the treated group, which indicates the non-toxicity of *omidun* treatment in this group. This is in resonance with histopathological examination done by Berthi et al. [15] of anti-plasmodial effect of plant extracts from *Picrolemma huberi* and *Picramnia latifolia* where the liver of mice treated with *Picrolemma huberi* was observed to have a slight increase in hepatocyte nucleus size, binucleation, congestion and macrophages while the kidney had no visible damage. The chloroquine treatment for the curative group showed moderate centrilobular hepatocellular vacuolar degeneration in the liver of the animal; this also shows that chloroquine treatment was completely unable to ameliorate damage done in the liver by the parasite but it had little ameliorating effect as there was no necrosis recorded, in contrast to the untreated group that recorded severe diffuse cord atrophy and hepatocellular necrosis. The kidney of this group however exhibited tubular epithelial degeneration and necrosis, which indicates the toxicity of chloroquine, and this is likely a reason why withdrawal from chloroquine and shifting to another form of treatment is advised [33].

In the prophylactic group, the *omidun* treatment revealed moderate diffuse cord atrophy and centrilobular coagulation necrosis in the liver while the kidney had necrosis of tubular epithelial cells and interstitial inflammation. This could be linked to the damaging effect of the parasites as *omidun* was unable to clear parasites in this group. The chloroquine treatment revealed moderate centrilobular hepatocellular atrophy and accentuation of sinusoids in the liver while the kidney had no observable lesion. Okpok et al. [34] attributed the damage of organs recorded in that study to a feature of severe *Plasmodium* infection or a characteristic of tissues previously exposed to parasitic infection.

In the suppressive group, *omidun* treatment revealed diffuse atrophy of hepatocytes and cord in the liver while the kidney of the animal had no observable lesion. The chloroquine treatment revealed moderate diffuse hepatocellular atrophy and accentuation of sinusoids in the liver of the animal while the kidney had necrosis of tubular epithelial cells and interstitial inflammation. This agrees with the study carried out by Diwan et al. [35] where histological changes was observed in the organs of mice treated with saponin extracted from *Citrullus colocynthis* plant. *Omidun* and chloroquine treatment was able to suppress the upset of parasite in the blood stream of the animal, it is expected that there would be no damage in the liver caused by the parasite, therefore damage observed in the liver of the animal may have arisen from other factors. This hypothesis is confirmed by the presence of damage in the liver of the animals that were uninfected and untreated. The toxicity of *omidun* can be said to be very low as there was no observable lesion in the kidney of the animals in this group.

The uninfected group but administered with chloroquine had severe diffuse cord atrophy and hepatocellular necrosis in the liver with tubular epithelial necrosis and inflammation in the kidney. The group administered with *omidun* without infection showed that the liver had random hepatocellular vacuolar degeneration while the kidney had no visible lesion; this indicates lesser toxicity than that of chloroquine as there was no necrosis recorded in the liver and no observable damage in the kidney.

The uninfected and untreated group showed that the liver had moderate diffuse vacuolar degeneration of the hepatocytes while the kidney had no visible lesion. This implies that the damage observed in the liver of treated groups may not have resulted from either the *omidun* or chloroquine since the group that was not administered with any treatment still revealed damage in the liver which could be linked to other factors, such as stress, fatigue, nutrition, atmospheric condition, and immune status of animals.

## Conclusion

The anti-malarial activity of *omidun* in this study is from the whole substance (viable cells and metabolites). *Omidun* served as a vehicle that facilitated the acquisition of the antimicrobial peptides synthesized by lactic acid bacteria.

Considering the impressive activities of *omidun* in curing, suppressing and arresting parasitaemia progressions, it may be safe to say that *omidun* has good anti-malarial potency which can be further investigated and exploited for malaria management in the tropics and sub-tropics. For maximum efficacy, there must however be a regular and continuous consumption of *omidun* to boost anti-malarial activity in humans. Future studies could investigate the safety of *omidun* for a longer period of time.

## Data Availability

Not applicable.
